# Use of the FLEX 28 Dexamethasone-Eluting Cochlear Implant Electrode in Electric–Acoustic Stimulation: A Case Report

**DOI:** 10.3390/audiolres15050112

**Published:** 2025-09-08

**Authors:** Shin-ichi Usami, Yutaka Takumi, Hidekane Yoshimura, Shin-ya Nishio

**Affiliations:** 1 Department of Hearing Implant Sciences, Shinshu University School of Medicine, Matsumoto 390-8621, Japan; nishio@shinshu-u.ac.jp; 2 Department of Otorhinolaryngology-Head and Neck Surgery, Shinshu University School of Medicine, Matsumoto 390-8621, Japan; takumi@shinshu-u.ac.jp (Y.T.); yoshimura@shinshu-u.ac.jp (H.Y.)

**Keywords:** electric–acoustic stimulation, dexamethasone-eluting electrode, cochlear implant, mitochondrial m.1555A > G variant, impedance field telemetry, hearing preservation, structure preservation

## Abstract

**Background/Objectives**: During and after electric–acoustic stimulation (EAS) surgery (as well as regular cochlear implant surgery), the oral and/or intravenous administration of steroids is recommended to prevent acute inflammatory reactions and subsequent fibrosis. However, the effect does not last long. Therefore, with the hope of providing a sustained effect, a new dexamethasone (DEX)-eluting electrode (FLEX28 DEX) has recently been developed. **Methods**: A case study was performed at Shinshu University in February 2024 in which a DEX-eluting electrode array was utilized for a patient presenting with high-frequency hearing loss with a defined etiology (hearing loss due to a mitochondrial m.1555A > G variant). **Results**: Residual hearing was well preserved after EAS surgery, and post-operative impedance field telemetry was maintained at a very low level in contrast with a historical/retrospective control group (FLEX28 electrodes without DEX); therefore, it is expected that post-operative fibrosis will be minimized. Further, it was shown that the DEX-eluting electrode can also be applied to EAS. **Conclusions**: The DEX-eluting electrode was useful in maintaining post-operative impedance at a very low level, indicating that post-operative fibrosis could be minimized even after EAS surgery.

## 1. Introduction

Cochlear implantation (CI) is the most important and effective treatment for patients with severe-to-profound sensorineural hearing loss (HL). In addition, the indications for CI have been expanded to patients with ski slope-type HL with residual hearing in lower frequencies and severe-to-profound HL in higher frequencies. Von Ilberg et al. developed the concept of electric–acoustic stimulation (EAS) combining low-frequency stimulation using hearing amplification together with electrical stimulation in the high frequencies [1]. EAS has afforded great benefits to patients with residual hearing [2,3,4,5,6,7] and is now used as a standard treatment option for patients with ski slope-type HL.

Various factors have been reported to affect the post-operative hearing preservation (HP), such as surgical approach, electrode flexibility, electrode insertion speed, insertion depth angle, round window opening size, round window accessibility, steroid administration, implant age, degree of residual hearing, etiology of HL and cochlear duct length [8,9,10,11,12,13,14,15,16,17,18,19,20]. A round window approach is preferable as (1) less drilling reduces acoustic trauma, (2) it ensures electrode insertion into the Scala tympani, and (3) it preserves vestibular function [8]. Indeed, we first demonstrated that HP can be achieved even when the electrode is located under the basilar membrane [8].

In addition, steroid administration is an important factor for HP, with systemic administration used pre- and post-operatively and topical administration also used intra-operatively [21,22,23]. During and after EAS surgery (as well as regular CI surgery), the use of steroids is recommended to prevent acute inflammatory reactions and subsequent fibrosis [24]. Previous studies also described the clinical utility of intra-cochlear steroid administration using an inner ear catheter [25,26,27]. However, the effect is not long-lasting. Therefore, with the hope of providing a sustained effect, a new dexamethasone (DEX)-eluting electrode has recently been developed. The pharmacokinetics of the DEX-eluting electrode have been investigated in preclinical studies, which showed sustained release over many weeks [28,29,30,31]. In addition, several preclinical studies showed favorable outcomes for DEX in reducing electrode insertion trauma as assessed by HP, decreasing impedance change after CI insertion, changing sensory compound action potential, and decreasing fibrosis after electrode insertion, outer hair cell loss and proinflammatory cytokine expression [29,32,33,34,35,36,37]. A feasibility study (Phase I study) indicated that the post-operative impedance of the DEX-eluting electrodes (FLEX28 DEX) is lower and more stable compared to that of the conventional FLEX28 electrodes [38]. Additionally, the feasibility study showed the possibility of preserving residual hearing. To date, however, there have been no reports of the use of DEX-eluting electrodes in patients who meet the criteria for EAS. Here, we first report our experience using DEX-eluting electrodes in a patient with a defined etiology that met the indications for EAS.

## 2. Materials and Methods

### 2.1. Case

The patient was a 53-year-old female with progressive HL. She noticed her HL at around age 20 and visited the Department of Otorhinolaryngology at Shinshu University Hospital at the age of 52 due to insufficient hearing with hearing aids. Social health insurance-based genetic testing [39] for this patient was conducted in 2023. Genetic testing was performed as a part of research approved by the Shinshu University Ethical Committee (approval number: No. 717—8 March 2022). The study protocol for EAS surgery and assessment using FLEX28 DEX (MED-EL, Innsbruck, Austria) was approved by the Shinshu University Certified Review Board of Clinical Research (approval number: No. 23008—5 December 2023) and was conducted in accordance with the Declaration of Helsinki. Informed consent was obtained prior to enrollment in this study.

### 2.2. Control Subjects

A control group was used to understand the positioning of the data obtained from this case. We used two control subjects to compare the post-operative HP and post-operative impedance field telemetry (IFT) change. (1) For HP, we used the average pre-operative and 12-month post-operative audiograms of 22 EAS patients. All of the 22 cases received EAS surgery using conventional FLEX28 (without DEX-eluting function). The average age of control group 1 at cochlear implantation was 35.5 ± 19.1 y.o. (range from 8 to 65). Seven subjects were male and fifteen subjects were female. Five cases had *CDH23*-associated HL, two cases had *SLC26A4*-associated HL and one case each had *KCNQ4-*associated HL and *LOXHD1-*associated HL. We could not identify any genetic cause for 13 cases. All subjects in this control group fulfilled the criteria for conventional EAS (≤65 dB HL in 125, 250 and 500 Hz, ≥80 dB HL in 2000 Hz and ≥85 dB HL in 4000 and 8000 Hz) in terms of preoperative hearing level. (2) For post-operative IFT, IFT data from 15 patients using conventional FLEX28 electrodes (without DEX-eluting function) were used. The average age of control group 2 at cochlear implantation was 42.0 ± 27.1 y.o. (range from 1 to 82). Five subjects were male and ten subjects were female.

### 2.3. Clinical Evaluation

Evaluation of hearing thresholds was performed using pure-tone audiometry pre-operatively and at 1, 3, 6, and 12 months after EAS surgery. Audiometric thresholds at four frequencies (0.5, 1, 2, and 4 kHz) were used to calculate the pure-tone average (PTA). Hearing thresholds with EAS were evaluated using free-field audiometry at 1, 3, 6, and 12 months after EAS surgery. Speech audiometry, including the Japanese monosyllable perception test (iCI2004 monosyllable) in quiet conditions, representing 65dBSPL, and the Japanese word recognition test (iCI2004 word), representing 65dBSPL in S/N +10 dB conditions, was performed at 1, 3, 6, and 12 months after EAS surgery [40].

IFT was performed to measure electrode impedance (kΩ) for all 12 channels intra-operatively and at 1, 3, 6, and 12 months after EAS surgery. Impedance values were obtained using the MAESTRO software package (Version 9, MED-EL, Innsbruck, Austria). The severity of HL was classified into four categories: mild (PTA > 25 dB and ≤40 dB HL), moderate (>40 dB and ≤70 dB HL), severe (>70 dB and ≤90 dB HL), and profound (>90 dB).

## 3. Results

### 3.1. Clinical Details of the Patient

**Figure 1:**
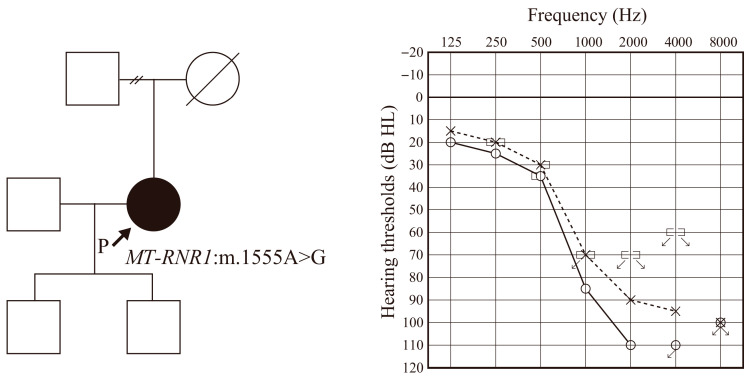
Pedigree, identified variant and audiogram of the patient. Squares indicate the male and circles indicate the female family members. Black indicates the affected individual and white indicates unaffected individuals. Solid line and dashed line indicates the hearing thresholds for right and left ear respectively.

The patient was a 53 y.o. female. She represented a case of sporadic HL without any other affected family members. Her audiogram showed precipitous high-frequency HL with residual HL in lower frequencies. Comprehensive genetic testing using next-generation sequencing identified the genetic cause of her HL as a mitochondrial m.1555A > G variant (Figure 1).

**Figure 2:**
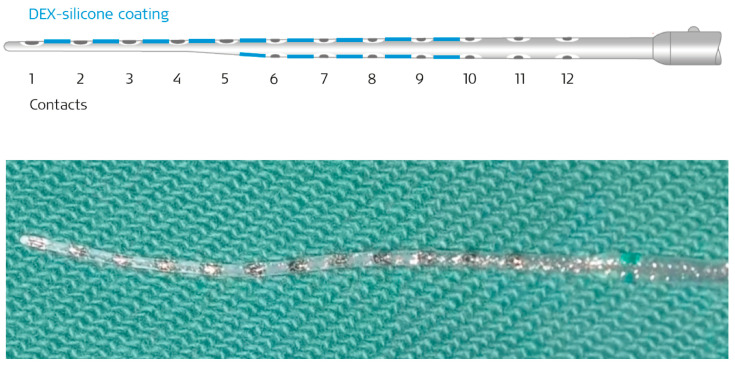
**Top**: Illustration of a FLEX28 DEX electrode array (MED-EL, Innsbruck, Austria); the blue areas represent the coating. **Bottom**: The white areas between the electrodes are coated with dexamethasone-containing silicone.

The patient wanted to receive EAS. With the hope of providing a sustained effect to reduce post-operative fibrosis surrounding the inserted electrode, and to preserve residual hearing at the lower frequencies, a new DEX-eluting electrode (FLEX28 DEX electrode array, MED-EL, Innsbruck, Austria) was used as part of a clinical trial in February 2024 (Figure 2).

### 3.2. Surgery and Implantation

**Figure 3:**
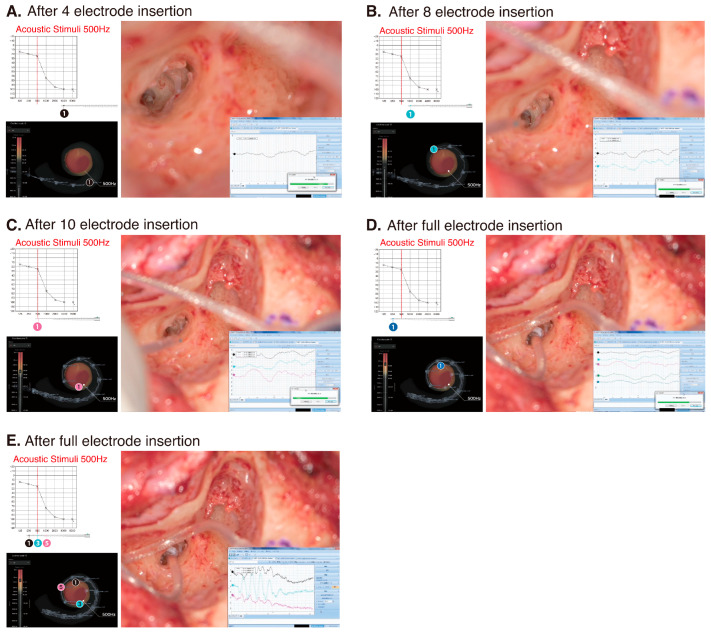
The exoscopic view of the surgical field, estimated electrode position in the audiogram and cochlear microphonics during electrode insertion. (**A**) The tip of the electrode has been inserted into the basal turn of the cochlear. As it is still far from the area responsible for the 500 Hz, no CM response is observed. (**B**) The first electrode has just reached the area responsible for 1000 Hz. A weak CM reaction is observed. (**C**) The first electrode has just reached the area responsible for 500 Hz. The maximum CM reaction has been obtained. (**D**) After full insertion, the first electrode is farther away from the area responsible for 500 Hz, so the response is weaker. (**E**) After full insertion, CM responses were taken from the first, third, and fifth electrodes, and the largest response was from the third electrode, which was located in the area responsible for 500 Hz. See Supplemental Materials (Video S1) for more high-resolution images.

The patient underwent a less-invasive surgical procedure for HP as described previously [7]. The round window membrane was widely exposed by removing the bony over-hang of the round window niche with a low-speed drill. Subsequently, the electrode was slowly inserted via the round window membrane. During electrode insertion, intraoperative cochlear microphonics (CM) monitoring was performed to confirm low-frequency functional preservation and to determine the most appropriate electrode position (Figure 3). During surgery, a 500 Hz acoustic stimulus was applied to the external auditory canal, and the CM response was recorded from the first electrode (Ch 1). The maximum response was obtained when the first electrode reached the area responsible for 500 Hz (Figure 3C). A video of the surgical procedure is available as Supplemental Materials.

### 3.3. Post-Operative Hearing Outcome and Impedance Change

**Figure 4:**
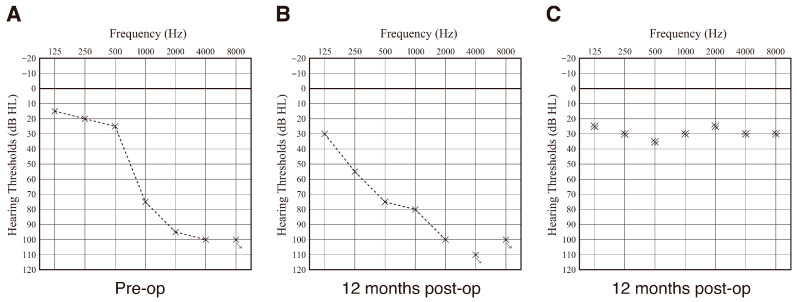
The audiogram and hearing level with EAS. (**A**) Audiograms taken pre-operatively and (**B**) at 12 months post-operatively, and (**C**) hearing level with EAS. Cross mark indicates the hearing threshold for each frequency.

**Figure 5:**
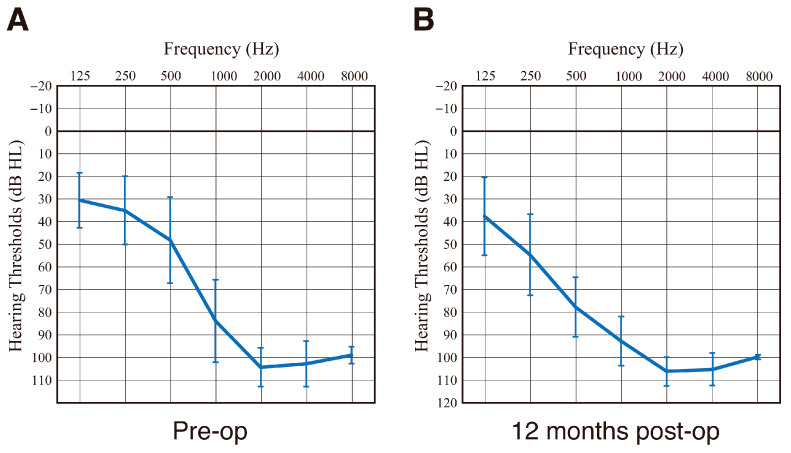
Averaged audiogram of 22 EAS patients using conventional FLEX28 (without DEX-eluting function). (**A**) Audiogram taken pre-operatively and (**B**) at 12 months post-operatively. Error bar indicates the standard deviation.

Based on the HP criteria [41], HP in this case was regarded as “partial (58.6%).” After electrode insertion, residual hearing available for acoustic stimulation was well preserved. Audiograms taken pre-operatively and at 12 months post-operatively are shown in Figure 4A,B. HP for this patient was comparable with that for 22 EAS patients using conventional FLEX28 electrodes (Figure 5). Hearing level with EAS is around 25–35 dB (Figure 4C). In addition, Japanese speech audiometry (iCI2004) also improved to 72% (monosyllable perception in quiet) and 92% (word recognition in SN + 10 dB).

**Figure 6:**
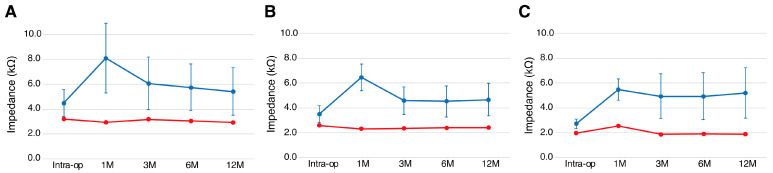
Post-operative impedance field telemetry (IFT) taken from (**A**) electrode Ch 1, (**B**) electrode Ch 5 and (**C**) electrode Ch 11. Blue indicates the average of 15 patients using conventional FLEX28 electrodes (without DEX). Red indicates the IFT for the patient with FLEX28 DEX. Error bar indicates the standard deviation of 15 controls. IFT was maintained at a very low level compared with the controls.

The impedance measured by IFT of the patient with the DEX-eluting electrode (FLEX28 DEX) was lower and quite stable compared to that of patients with the conventional FLEX28 electrode (Figure 6). Therefore, it is expected that post-operative fibrosis will be minimized.

## 4. Discussion

Pre- and post-operative systemic and/or topical steroid administration is an important factor in facilitating HP [21,22,23,24]. In addition, intra-cochlear steroid administration is also performed via an inner ear catheter [25,26,27]. A double-blinded placebo-controlled trial for intra-operative systemic methylprednisolone has been performed, but no statistical differences were observed between the treatment and placebo control groups [42]; however, several clinical papers report positive outcomes for pre- and post-operative systemic and/or topical steroid administration and such steroid administration is recommended. To provide sustained steroid administration, the DEX-eluting electrode was developed. Several preclinical studies showed the DEX-eluting electrode to be useful in reducing electrode insertion trauma as assessed by HP, decreasing impedance change after CI insertion, changing compound action potential, and decreasing fibrosis after electrode insertion, outer hair cell loss and proinflammatory cytokine expression [29,32,33,34,35,36,37]. Intracochlear sustained dexamethasone release in combination with CI in two patients has been reported as a proof of concept with regard to the reduction of impedance [43]. The sustained dexamethasone release from the electrode is also useful in maintaining lower impedance after CI [44]. Recently the results of a phase I study have been reported, with the post-operative impedance of the DEX-eluting electrodes (FLEX28 DEX) shown to be lower and more stable compared to that of conventional FLEX28 electrodes [38].

The mitochondrial m.1555A > G variant is one of the most prevalent genetic causes of maternally inherited HL among the pathogenic variants in mitochondrial DNA that is identified in different populations [39,45,46,47,48,49,50,51,52]. Additionally, it is recognized to increase the susceptibility to aminoglycoside antibiotics and the risk of aminoglycoside-induced HL [45,52,53]. In general, aminoglycoside-induced HL is bilateral and severe-to-profound in all frequencies, occurring within several days after the administration of aminoglycoside antibiotics [45]. However, HL is sometimes seen in patients with no known history of aminoglycoside administration, although in these cases the HL is usually milder and involves higher frequencies [45,52]. Mitochondria are known as organelles that produce energy (adenosine triphosphate; ATP) necessary for hearing, but mutated mitochondria show a decrease in energy production. As a result, there is a lack of energy to convert sound vibrations to electrical signals, leading to HL. As the latter group of patients usually have residual hearing at the lower frequencies, they are good candidates for EAS [54,55].

**Figure 7:**
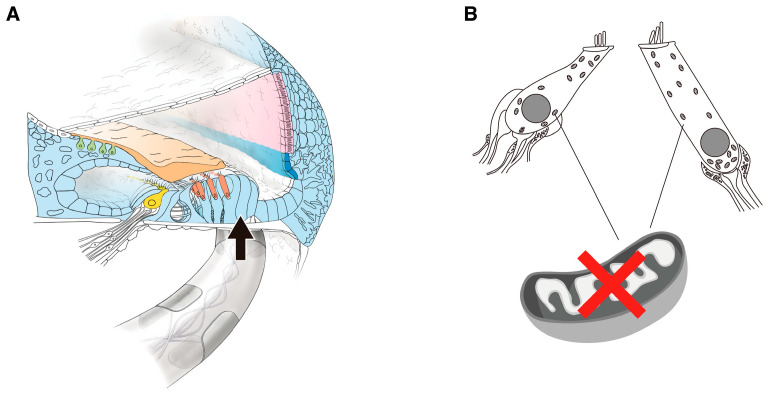
Possible phenomena occurring within the cochlea during electrode insertion in patients with the mitochondrial m.1555A > G variant. (**A**) Inserted electrodes touch basilar membrane and inhibit its movement. (**B**) Inflammation induced by electrode insertion in addition to the lack of energy caused by mitochondrial mutation can lead to even more severe hearing deterioration.

Understanding the etiology allows us to imagine what is happening inside the cochlea. With regard to possible phenomena occurring in the cochlea during electrode insertion in patients with the mitochondrial m.1555A > G variant, the following factors may result in HL due to electrode insertion: (1) electrodes inhibit the movement of the basilar membrane, and (2) mutated mitochondria cause a decrease in energy production. If inflammation induced by electrode insertion is added to this condition, the lack of energy can lead to even more severe hearing deterioration. For the second factor, DEX-eluting electrodes can be used to prevent reactions mediated by inflammation (Figure 7).

With regard to the residual hearing at lower frequencies, many factors, including age at which EAS was performed, individual cochlear volume, electrodes used, and surgical method, have been reported to be involved [8,9,10,11,12,13,14,15,16,17,18,19,20]. Of these, damage occurs within the cochlea and hearing deteriorates with the use of rigid electrodes or when cochleostomy or the extended RW approach is used. However, when flexible electrodes are used and inserted via the RW approach, there may be individual differences in HP due to differences in the position of the electrode for each case (first factor) and differences in the pathological inflammatory response (second factor) mentioned above. Of course, there is potential for inner ear trauma and deteriorated residual hearing as a result of a pathological inflammatory response.

In this clinical case, the residual hearing available for acoustic stimulation was well preserved (Figure 4). HP was comparable to that of the 22 EAS patients using conventional FLEX28 electrodes (Figure 5). Intraoperative CM monitoring was found to be useful for HP (i.e., monitoring whether outer hair cells are viable) and for confirming optimal electrode positioning. The maximum response was obtained when the first electrode reached the area responsible for 500 Hz, proving that these monitoring techniques were performed accurately [56,57]. The concept underlying HP includes minimizing operation-associated trauma and enhancing the preservation of cochlear structures, which is important in expanding future treatment strategies such as gene therapy or regeneration therapy.

The most remarkable result was the change in post-operative impedance over time. As shown in Figure 6, the impedance of the DEX-eluting electrode (FLEX28 DEX) is very low and more stable compared to that of the conventional FLEX28 electrode. As the impedance (resistance value when a current is passed through the electrode) is believed to be a marker of post-operative fibrosis surrounding the inserted electrode [35], it is expected that post-operative fibrosis will be minimized and that there will be a positive long-term effect on hearing performance with CI/EAS. The fact that the DEX-eluting electrode also had a favorable effect on EAS in this study suggests that it can also be applied (instead of conventional electrodes) for all patients in the near future.

## 5. Conclusions

In this study, we clearly showed the clinical utility of DEX-eluting electrodes, particularly in terms of achieving lower and more stable post-operative impedance. Our results indicate an expected reduction in post-operative fibrosis surrounding the inserted electrode. Thus, the DEX-eluting electrodes can be applied (instead of conventional electrodes) for all CI/EAS patients in the near future.

## Data Availability

The original contributions presented in this study are included in the article/supplementary material. Further inquiries can be directed to the corresponding author.

## Supplementary Materials

The following supporting information can be downloaded at https://www.mdpi.com/article/10.3390/audiolres15050112/s1, Video S1: Surgical procedure of FLEX28 DEX electrode for EAS.
